# Transcriptomic Analysis of Laying Hens Revealed the Role of Aging-Related Genes during Forced Molting

**DOI:** 10.3390/genes12111767

**Published:** 2021-11-07

**Authors:** Tongyu Zhang, Yu Chen, Junhui Wen, Yaxiong Jia, Liang Wang, Xueze Lv, Weifang Yang, Changqing Qu, Haiying Li, Huie Wang, Lujiang Qu, Zhonghua Ning

**Affiliations:** 1 State Key Laboratory of Animal Nutrition, Department of Animal Genetics and Breeding, National Engineering Laboratory for Animal Breeding, College of Animal Science and Technology, China Agricultural University, Beijing 100193, China; zhangty0611@126.com (T.Z.); wjh8545@cau.edu.cn (J.W.); quluj@cau.edu.cn (L.Q.); 2 Beijing Animal Husbandry and Veterinary Station, Beijing 100107, China; chenyu.cncn@163.com (Y.C.); wangliangcau@139.com (L.W.); lvxueze0310@163.com (X.L.); carspstp@126.com (W.Y.); 3 Institute of Animal Science, Chinese Academy of Agricultural Sciences, Beijing 100193, China; jiayaxiong@caas.cn; 4 Engineering Technology Research Center of Anti-Aging Chinese Herbal Medicine of Anhui Province, Fuyang Normal University, Fuyang 236037, China; qucq518@163.com; 5 College of Animal Science, Xinjiang Agricultural University, Urumqi 830052, China; lhy-3@163.com; 6 College of Animal Science, Tarim University, Alar 843300, China; whedky@126.com

**Keywords:** forced molting, aging, rejuvenation, transcriptome analysis, hens

## Abstract

Molting in birds provides us with an ideal genetic model for understanding aging and rejuvenation since birds present younger characteristics for reproduction and appearance after molting. Forced molting (FM) by fasting in chickens causes aging of their reproductive system and then promotes cell redevelopment by providing water and feed again. To reveal the genetic mechanism of rejuvenation, we detected blood hormone indexes and gene expression levels in the hypothalamus and ovary of hens from five different periods during FM. Three hormones were identified as participating in FM. Furthermore, the variation trends of gene expression levels in the hypothalamus and ovary at five different stages were found to be basically similar using transcriptome analysis. Among them, 45 genes were found to regulate cell aging during fasting stress and 12 genes were found to promote cell development during the recovery period in the hypothalamus. In addition, five hub genes (*INO80D, HELZ, AGO4, ROCK2,* and *RFX7*) were identified by WGCNA. FM can restart the reproductive function of aged hens by regulating expression levels of genes associated with aging and development. Our study not only enriches the theoretical basis of FM but also provides insights for the study of antiaging in humans and the conception mechanism in elderly women.

## 1. Introduction 

Molting is a natural and energy-demanding life history process of birds for adaptation to complex environmental changes [1]. It usually takes 14–16 weeks to complete the molting process, and the egg-laying rate (ELR) gradually decreases under natural conditions in chickens. The ELR here refers to the proportion of the total number of eggs laid by 100 hens of the same day age in one day. However, forced molting (FM) usually requires only 4 weeks to complete. Therefore, FM can achieve centralized and unified molting over a short period of time, which is convenient for production management and can improve the ELR and egg quality after molting. Thus, to shorten molting time and improve economic benefits, laying hens are often subjected to FM [2].

FM refers to the protocol that artificially imposes stress factors on hens, which cause unique morphological and physiological changes in their reproductive system, so that the hens stop laying and lose feathers and weight over a short period of time. They then grow new feathers and reach the second peak of egg production (SPEP), reproducing for another year after FM. FM is applied using various methods, such as fasting or feeding with a high-zinc diet. However, the fasting method is the most popular method for farmers because of its simplicity [3]. 

The laying hens are subjected to intense stress by artificial control measures. Intense stress causes the lack of cellular calcium [4,5], which is the main substance that maintains the secretion of the neurohormone, and the lack of calcium will affect the regulatory function of the hypothalamus [6,7,8]. To adapt to the stress of starvation, the hypothalamic–ovarian–gonadal axis plays an important role in regulating the liver lipid metabolism and maintaining the stability of blood sugar based on the role of the thyroid gland or sex hormone [9]. However, a lack of calcium inhibits the secretion of pituitary luteinizing hormone (LH), resulting in a decrease in plasma LH level or even cessation of estrogen secretion. Consequently, hens decrease in weight and immunity [10], the oviduct isthmus degenerates [11,12], and ovarian aging and atrophy occur [13], resulting in reduced ELR and eggshell quality.

When the stress is relieved by resupplying water, chickens meet the energy requirements of the body, reactivating nerves and endocrine function [14]. The survival potential of laying hens is gradually recovered during this process. The most obvious manifestations are the regrowth of new feathers, decreased mortality rate, improved eggshell quality [15,16,17,18], and gradual recovery of ELR to SPEP, which is higher than the premolting level [2]. Additionally, the abundance of bacteria in the feces [19] and bone strength [20,21] are also higher than before. Therefore, FM of hens is of substantial importance to increase the utilization life of aging hens.

At present, FM by fasting is increasingly widely applied, but its genetic mechanism has not been elucidated. With the development of next-generation sequencing technology, the transcriptome is highly accurate in the detection of differentially expressed genes (DEGs) for important economic traits in chickens, such as growth and development [22,23,24,25], reproduction traits [26,27,28,29], and disease-resistance traits [30,31,32,33]. However, a transcriptome study of FM has not yet been conducted in chickens. However, on the one hand, numerous studies have shown that intermittent fasting is healthy for human and animal health, which can delay cell aging, promote autophagy, and enhance immunity [34,35,36]. On the other hand, an increasing number of older women are having trouble conceiving. Therefore, we hypothesized that the genetic mechanism of FM by fasting in chicken would be like the above two cases. Consequently, we performed a transcriptome analysis to reveal the dynamic expression mechanism of regulatory genes during FM in laying hens. Furthermore, we determined whether our research could provide a reference for research on the mechanism of human antiaging and the conception mechanism of elderly women. 

## 2. Materials and Methods

### 2.1. Ethics Statement 

All experiments were approved by the Committee for Animal Care and Use of China Agricultural University (Approval ID: XXCB-20090209). The experimental procedures using chickens were performed according to the Guidelines for Experimental Animals established by the Ministry of Science and Technology (Beijing, China).

### 2.2. Animal Experimental Design 

**Figure 1:**
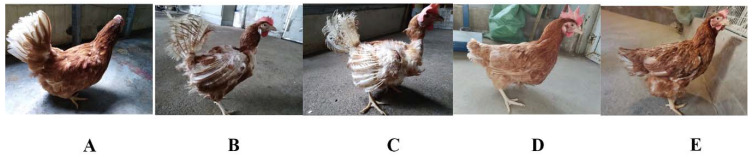
Feather appearance phenotypes in hens of five different growth periods during FM. Period 1 (**A**), Period 2 (**B**), Period 3 (**C**), Period 4 (**D**), Period 5 (**E**).

A total of 44,079 Jingfen No. 6 laying hens were selected to perform FM at the laying farm of Hubei Shendan Company (Anlu, China). All chickens were kept in a six-story coop under closed management. The experimental chickens were divided into five different periods according to their growth period (Figure 1, Table 1). The 224-day-old chickens were under the first peak of egg production, and the ELR was 0.941 (period 1). The ELR of 456-day-old chickens before molting was decreased to 0.774 (period 2), indicating that artificial FM could be conducted to increase the ELR again. All chickens were injected with vitamins to boost immunity during the first 12 days; moreover, the sick and dead chickens were eliminated in a timely manner. During the 12-day fast, laying hens were not fed, and only stones were provided on the fifth day. The chickens were not supplied with water for the first two days, but the water was not normally supplied until the third day. The chickens were then gradually cut off from water and feed supply for 10 days until the chickens lost 30% of their body weight. During this time, the chicken feathers gradually fell off, the daily death rate increased, and the ELR of 469-day-old hens declined to 0.002 (period 3). Then, water and feed were supplied gradually. At this time, the chickens progressively recovered their early normal condition with increases in weight, feathers, and ELR. The ELR of 500-day-old hens was restored to half of its previous level and was 0.472 (period 4). Finally, the ELR of the 527-day-old hens was up to 0.873 and remained for a period of time, belonging to SPEP (period 5).

Three healthy laying hens with uniform body weight were selected as experimental hens in five periods (Table 1), and blood and tissue samples were collected after slaughter at the same time.

### 2.3. Serological Indices

Blood samples from 15 chickens were incubated overnight at 4 °C. Serum was separated by centrifugation at 20,000× g for 5 min in anticoagulant tubes. Thyroxine (T4), progesterone (PROG), estrogen (E), thyroid-stimulating hormone (TSH), follicle-stimulating hormone (FSH), calcitonin (CT), and growth hormone (GH) levels were then measured by enzyme-linked immunosorbent assay (ELISA) at the Bioygene Biological Technology Co., Ltd. (Wuhan, China). Student’s t-tests were used to analyze the differences in serological indices between two periods.

### 2.4. Sample Collection

The chicken skull was opened, and 100–200 mg of hypothalamic tissue was removed. Then, the chicken’s abdomen was dissected, ovarian tissue was removed, and 200 mg of ovarian tissue was isolated. Tissue samples were washed in 0.9% normal saline, placed in RNAseq-free storage tubes, frozen in liquid nitrogen, and stored at −80 °C.

### 2.5. RNA Isolation, Library Construction, and Sequencing

Total RNA from chicken hypothalamic and ovarian tissue was extracted using TRIzol reagent (Invitrogen, Carlsbad, CA, USA). The RNA concentration was measured using a NanoDrop ND2000 spectrophotometer (NanoDrop Products, Wilmington, DE, USA), according to the manufacturer’s instructions. Agilent 2100 Bioanalyzer (Agilent Technologies, Santa Clara, CA, USA) was used to detect integrity and concentration of the extracted RNA using agarose gel electrophoresis.

Then, high-quality RNA samples (concentration >50 ng/μL, OD260/280 = 1.8 ∼ 2.2, OD260/230 = 1.8 ∼ 2.2, RIN > 8, 28S:18S ≥ 0.5) were used to construct sequencing libraries. Poly (A) mRNA was isolated from the total RNA samples with oligo (dT) magnetic beads (Invitrogen), and the RNA was broken to a fragment of about 300 bp in length by means of ion breaking. The first cDNA strand was synthesized with 6-base random primers and reverse transcriptase, and the second cDNA strand was synthesized with the first cDNA strand as template synthesis of cDNA using RNA as template. Libraries were size selected for cDNA target fragments of 450bp, followed by PCR amplification. Finally, the 30 libraries of the hypothalamus and ovary were sequenced using Illumina HiSeqTM2500 (150 bp paired-end reads) at Shanghai Personal Biotechnology Co., Ltd. (Shanghai, China).

### 2.6. Alignment with the Reference Genome and DEGs Analysis

Raw FASTQ data were first checked for high quality using fastp software with default parameters to remove joints, blank reads, and low-quality sequences (sequences with N ratio > 10% and Q-value < 20%). Then, high-quality clean reads were obtained for subsequent analysis. Then HISAT2-2.2.0 software was applied to map clean reads to the reference genome Gallus_gallus-6.0 (https://www.ncbi.nlm.nih.gov/assembly/GCF_000002315.6, accessed on 20 November 2020). Gene expression quantification was performed using normalized numbers of FPKM method. Then, we performed RNA-seq analysis using hypothalamus and ovarian tissues from five periods with the DESeq R package. DEGs were finally selected using a cutoff at false discovery rate < 0.05 and |log2FoldChange| ≥ 1.

### 2.7. Functional Annotation of DEGs

GO analysis is a commonly used approach for defining genes and their RNA or protein products by identifying unique biological properties of high-throughput transcriptome or genome data. Based on the GO terms of transcripts, these DEGs were assigned into three main functional categories: biological process (BP), cellular component (CC), and molecular function (MF). KEGG is a collection of databases dealing with genomes, diseases, biological pathways, and chemical materials. DAVID, which is an online bioinformatics tool, is designed to identify numbers of genes or protein functions. We used DAVID to visualize DEG enrichment of BP, MF, CC, and pathways (*p*  <  0.05).

### 2.8. Trend Analysis

Gene expression pattern analysis was used to cluster genes with similar expression patterns for multiple samples (at least three in a specific time point, space, or treatment dose-size order). To examine the expression pattern of DEGs, the expression data of each tissue sample were normalized to 0, log2 (v1/v0), and log2 (v2/v0) and then clustered using Short Time-Series Expression Miner (STEM) software (http://www.sb.cs.cmu.edu/stem/, accessed on 10 February 2021). The parameters were set as follows:

(1) Maximum unit change in model profiles between time points was 1;
(2) Maximum output profile number was 20 (similar profiles will be merged);
(3) Minimum ratio of fold change of DEGs was no less than 2.

The clustered profiles with *p*-value ≤ 0.05 were considered significant profiles. To understand the functions of the DEGs in each profile, GO and KEGG pathway enrichment analyses were performed using DAVID. The GO terms or pathways with a Q-value ≤ 0.05 were defined as significantly enriched GO terms or pathways.

### 2.9. Construction of Gene Coexpression Networks and Screening of Hub Genes 

WGCNA was used to analyze coexpression of genes and select highly correlated genes (hub genes) that may be strongly associated with complex life activities related to FM. The genes with very low expression values were not considered in this analysis to avoid the inclusion of spurious edges in the networks. After completion of the coexpression analysis, the edge files were sorted by weight, and the first 100 pairs of network connections were used to establish interaction networks among the DEGs. The hub genes were screened based on the module membership (K_ME_) values. The interaction networks were drawn using Cytoscape 3.7.1.

## 3. Results

### 3.1. Comparison of Changes in Feather Coverage during FM 

During the five periods of FM, the feather coverage of laying hens varied greatly (Figure 1, Table 1). Feathers tend to fall off with age (Figure 1A,B), and hunger stress reinforces this trend in the short term (Figure 1B,C). With the water and feed supply, the laying hens regrow new feathers, and the color is beautiful, looking younger (Figure 1C–E). Therefore, we hypothesize that numbers of hormones and genes may be involved in the regulation of this complex and interesting rejuvenating phenomenon.

### 3.2. Serological Indices between Two Periods in the Five FM Periods 

To explore the trend in variation of related serological indexes in chickens during FM, we tested seven serological indices, namely thyroxine (T4), progesterone (PROG), estrogen (E), thyroid-stimulating hormone (TSH), follicle-stimulating hormone (FSH), calcitonin (CT), and growth hormone (GH), by ELISA [37] (Table S1). The results showed that only E in the 2-vs-3 group (*p* = 0.029), GH in the 3-vs-4 group (*p* = 0.044), and TSH in the 4-vs-5 group (*p* = 0.023) comparisons were significantly different (*p* < 0.05) (Table S1).

### 3.3. RNA-Seq Analysis for Identifying DEGs among Three Groups 

**Figure 2:**
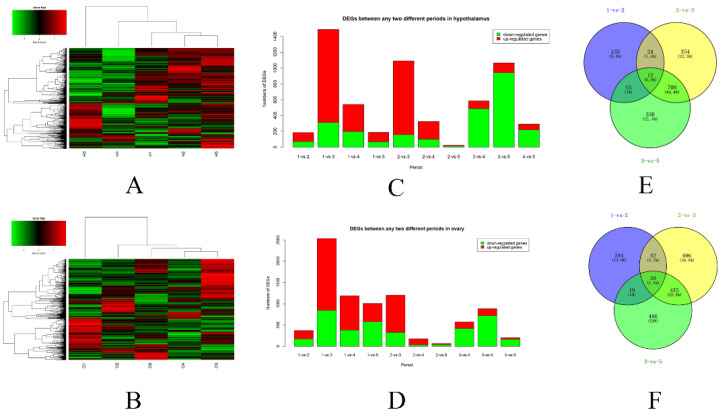
Transcriptome analysis of hypothalamus and ovary in 5 periods during FM. Overall profiles of mRNA expression in the hypothalamus (**A**) and ovary (**B**). DEGs between any two different periods in the hypothalamus (**C**) and ovary (**D**). Venn diagram shows the DEGs from the 1-vs-2, 2-vs-3, and 3-vs-5 comparisons in the hypothalamus (**E**) and ovary (**F**).

The clean reads of the 30 samples were all high-quality with Q20 > 96% and Q30 > 92%, and the GC content was approximately 50% (Table S2). There were 24,128 detectable genes expressed in both the hypothalamus and ovary. A heatmap and hierarchical clustering were established for all the detectable genes in the five periods, with average fragments per kilobase per million mapped reads (FPKM) values for the hypothalamus (Figure 2A) and ovary (Figure 2B). Overall, the expression levels of the same genes in different periods were different. Additionally, the expression levels in periods 1, 2, and 5 in the hypothalamus and ovary were similar. The DEGs (including upregulated and downregulated genes) in the hypothalamus (Figure 2C) and ovary (Figure 2D) were varied among groups (Table S3). The DEGs in the groups of 2-vs-5 were relatively fewest, whereas there were more DEGs in the groups of 1-vs-2, 2-vs-3, and 3-vs-5 in both the hypothalamus and ovary. Therefore, to further explore the dynamic gene expression pattern during the FM of laying hens, we focused on the DEGs from the 1-vs-2, 2-vs-3, and 3-vs-5 groups in both the hypothalamus and ovary. The Venn diagram shows the distribution of DEGs in the three groups in the hypothalamus (Figure 2E) and ovary (Figure 2F), and 12 and 28 genes were shared among the three groups, respectively. The DEGs in the 2-vs-3 and 3-vs-5 groups were much more similar, with 45.2% common DEGs (712) in the hypothalamus (Figure 2E) and 24.3% common DEGs (463) in the ovary (Figure 2F). 

### 3.4. Functional Enrichment and Annotation of DEGs among the Three Groups 

**Figure 3:**
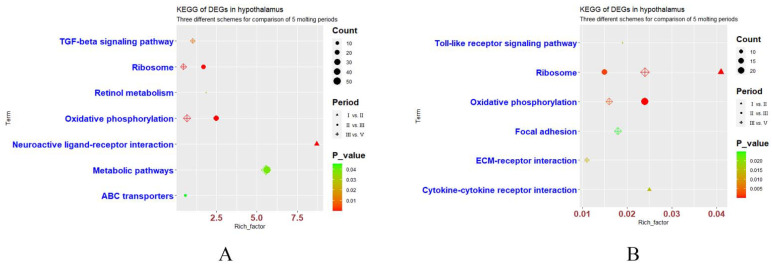
The KEGG pathways of three groups of DEGs in hypothalamus and ovary during FM. Significant KEGG pathways of DEGs (*p* < 0.05) from the comparison of Ⅰ vs. Ⅱ, Ⅱ vs. Ⅲ, and Ⅲ vs. Ⅴ in the hypothalamus (**A**) and ovary (**B**).

To explore the relevant biological functions of DEGs, all the DEGs of the three groups (1-vs-2, 2-vs-3, and 3-vs-5) were analyzed by gene ontology (GO) term enrichment (Figure S1–S6) and Kyoto Encyclopedia of Genes and Genomes (KEGG) pathways (Figure 3). 

#### 3.4.1. Group 1-vs-2 in the Hypothalamus and Ovary

In our study, a total of 184 DEGs from the 1-vs-2 group in the hypothalamus were used for GO term enrichment analysis (Additional file 1). Twenty GO terms (11 BP, 2 CC, and 7 MF) (Figure S1) and two KEGG pathways (Figure 3A) showed significant differences (*p* < 0.05) (Additional file 2). GO terms were enriched in the regulation of retina homeostasis, response to heat, and embryonic skeletal system morphogenesis. KEGG pathways were enriched in neuroactive ligand–receptor interactions and retinol metabolism.

A total of 373 DEGs in the ovary were used to analyze GO terms and KEGG pathway analyses (Additional file 3). A total of 14 GO terms (6 BP, 6 CC, 2 MF) (Figure S2) and 3 KEGG pathways (Figure 3B) showed significant difference (*p* < 0.05) (Additional file 4). GO terms were enriched in immune response, translation, cell chemotaxis, cell surface receptor signaling pathway, myeloid dendritic cell differentiation, and cell projection assembly. Additionally, three significant KEGG pathways were ribosome, cytokine–cytokine receptor interaction, and toll-like receptor signaling pathway.

#### 3.4.2. Group 2-vs-3 in the Hypothalamus and Ovary

In the 2-vs-3 group, a total of 1090 DEGs in the hypothalamus were used to perform GO term and KEGG pathway analyses (Additional file 1). There were 27 significant GO terms (13 BP, 6 CC, 8 MF) (Figure S3) and 4 significant KEGG pathways (Figure 3A) (*p* < 0.05) (Additional file 2). The 27 GO terms were enriched in protein refolding, translation, and neuropeptide signaling pathway, and the four significant KEGG pathways were oxidative phosphorylation, ribosome, metabolic pathways, and ABC transporters.

A total of 1201 DEGs in the ovary were used to perform GO term and KEGG pathway analyses (Additional file 3). Thirty-three significant GO terms (22 BP, 7 CC, 4 MF) (Figure S4) and two KEGG pathways (Figure 3B) were obtained (*p* < 0.05) (Additional file 4). GO terms were enriched in face morphogenesis, chemical synaptic transmission, and dopamine biosynthetic process. Two significant KEGG pathways were enriched in oxidative phosphorylation and ribosomes.

#### 3.4.3. Group 3-vs-5 in the Hypothalamus and Ovary

A total of 1065 DEGs from the hypothalamus of the 3-vs-5 group were used to perform GO term and KEGG pathway analyses (Additional file 1). Twenty-three significant GO terms (12 BP, 3 CC, 8 MF) (Figure S5) and four KEGG pathways (Figure 3A) were obtained (*p* < 0.05) (Additional file 2). GO terms were enriched in translation, multicellular organism development, and negative regulation of osteoblast differentiation. The four significant KEGG pathways were oxidative phosphorylation, ribosome, TGF-β signaling pathway, and metabolic pathways.

Additionally, a total of 882 DEGs in the ovary during this period were used to perform GO term and KEGG pathway analyses (Additional file 3). There were 27 significant GO terms (8 BP, 10 CC, 9 MF) (Figure S6) and 4 significant KEGG pathways (Figure 3B) (*p* < 0.05) (Additional file 4). GO terms were enriched in translation, skeletal muscle thin filament assembly, and calcium ion-regulated exocytosis of neurotransmitters. The four significant KEGG pathways were ribosome, oxidative phosphorylation, ECM–receptor interaction, and focal adhesion.

### 3.5. Short Time-Series Expression Miner Analysis of Hypothalamus and Ovary

**Figure 4:**
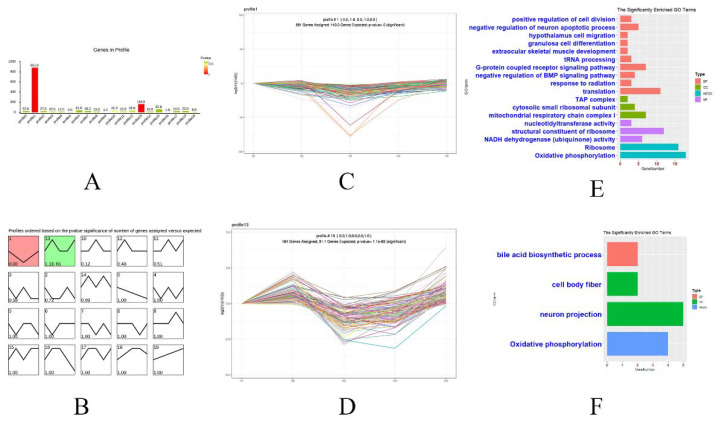
All the DEGs in the hypothalamus and ovary of the 3 groups were analyzed by Short Time-Series Expression Miner (STEM). STEM clustering of the numbers of DEGs at 19 profiles in the hypothalamus (**A**). The number on the top right corner represents the cardinality of each cluster, and the number on the bottom left represents the adjusted *p*-value; all significant profiles based on *p* values of the numbers of genes (**B**). Expression patterns of profiles 1 (**C**) and 13 (**D**). GO analysis (BP, CC, and MF) and KEGG pathways of genes clustered in profiles 1 (**E**) and 13 (**F**).

To illustrate the dynamic gene expression pattern during hypothalamus and ovary development, we normalized the expression data of DEGs to those of period 1 (control) and determined the temporal gene expression profiles using STEM. A total of 1473 FPKM in the hypothalamus and 1830 FPKM in the ovary for all DEGs from the above three groups (1-vs-2, 2-vs-3, and 3-vs-5) were used as raw data (Figure 2E,F). The normalized data for each period were the average value of the three sets of biological duplicate data. The profile boxes depicted gene expression patterns over the five periods (Figure 4B). Ultimately, only profiles 1 (881 genes) and 13 (164 genes) were statistically significant within the 20 model profiles (Figure 4A,B) (*p* < 0.05). Profile 1 exhibited biphasic responding expression patterns (Figure 4B,C), whereas profile 13 gradually increased during period 2 and then decreased during period 3, remained stable during the 3–4 period, and increased gradually at later time points (Figure 4B,D). Genes in profiles 1 and 13 are summarized in Additional file 5. To excavate the gene functions, GO terms and KEGG pathways (profiles 1 and 13) were analyzed using DAVID (https://david.ncifcrf.gov/, accessed on 25 February 2021).

Genes in profile 1 were associated with 16 GO terms, such as extraocular skeletal muscle development, hypothalamic cell migration, and positive regulation of cell division. KEGG pathways were enriched only for oxidative phosphorylation and ribosomes (Figure 4E). However, there were only three significant GO terms (bile acid biosynthetic process, neuron projection, and cell body fiber) and one KEGG pathway (oxidative phosphorylation) enriched in genes of profile 13 (Figure 4F). 

Overall, we detected 322 genes in profile 1 whose expression trends were consistent with ELR (Additional file 5). Among them, 45 genes contributed to cell senescence, 8 were involved in autophagy in cells and organs when their expression was inhibited, and 12 promoted the development of cells and organs when their expression was increased (Table S4). 

### 3.6. Weighted Gene Coexpression Network Analysis of the Hypothalamus and Ovary

Because the hypothalamus and ovaries played an important role in the entire process of FM, we combined the expression profiles of the hypothalamus and ovary in the original data containing 30 samples for weighted gene coexpression network analysis (WGCNA). We used normalized expression values of 18,196 genes from 30 samples of the hypothalamus and ovary to construct the coexpression module. The expression profile of the top 8000 genes passed the good gene or good sample tests. Thus, the phenotypic data included five periods and two tissues. All the previous analyses were based on the WGCNA R package. 

#### 3.6.1. Construction of Coexpression Modules of FM

**Figure 5:**
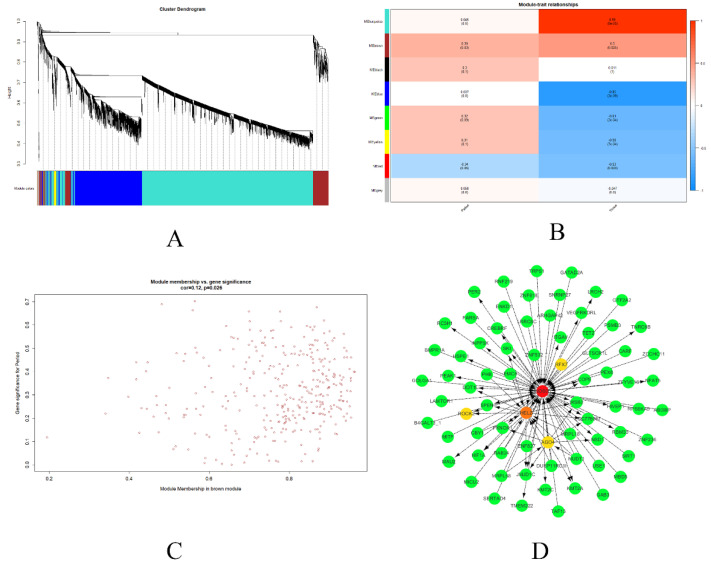
The data of all genes expressed in the hypothalamus and ovary in 5 periods were analyzed by WGCNA. Gene dendrogram obtained based on a dissimilarity measure (1-TOM) (**A**). Relationships of module eigengenes vs. period and tissue (**B**). Scatterplots of gene significance (GS) vs. module membership (MM) in the brown module (**C**). Visualization of modules: the hub genes in the modules are highlighted in red and yellow (**D**).

When the soft-thresholding power was equal to 8 (power β = 8), the correlation coefficient reached 0.8, and a scale-free coexpression network was achieved (Figure S7). Next, the power value was used to construct the coexpression module, and the results showed that seven distinct gene coexpression modules were identified during FM. These coexpression modules were constructed and shown in different colors (Figure 5A). 

#### 3.6.2. Gene Coexpression Modules Correspond to Phenotypic Traits

We only focused on changes in gene expression during five periods of FM. Modules with common expression patterns in interaction analysis of coexpression modules associated with specific traits (module–trait) were identified based on the correlation between module eigengenes and phenotypic traits. The correlation of modules and traits related to the five periods (module–trait) showed that some modules were more important than others in the periods. The brown-colored module had the greatest association with period traits (Figure 5B). Therefore, the brown module, including 342 genes, should be captured, and it summarized the most mRNA expression profiles. Scatter diagrams of gene significance for period vs. module membership in the brown modules were plotted (Figure 5C), which exhibited the significance of the genes in the brown module relative to different periods (*p* = 0.026). 

#### 3.6.3. Module Visualization and Hub Genes

The top 100 genes in the brown modules were used in Cytoscape software, and intramodular connectivity was calculated. Intramodular connectivity was calculated for each gene by summing the connection strengths with other module genes and dividing this number by the maximum intramodular connectivity. Genes with high intramodular connectivity were considered intramodular hub genes. The top five genes in the network of the top 100 brown genes were ranked using maximal clique centrality (MCC) algorithm, and the top five genes (Table S5) are highlighted in red or yellow (Figure 5D).

## 4. Discussion

### 4.1. Fasting Promotes the Health of Both People and Animals 

With the improvement of substance levels, some people become addicted to binge eating, which not only brings harm to their physical health but also leads to emotional disorders [38]. Fasting has been reported as beneficial in maintaining weight and preventing disease [39,40]. Moreover, caloric restriction, time-restricted feeding, and intermittent fasting (IF) can eliminate damaged organelles by autophagy of tissues and organs in response to food deprivation to slow aging and increase longevity [41]. FM of chickens in our study was a biological process of rejuvenation through restriction of diet or fasting. After FM, laying hens regrew new feathers and became healthier than before, additionally, ELR of chickens generally recovered to the level of the first peak, and both egg quality and eggshell quality were improved [42]; even the gut microbiota was richer and improved feed efficiency [19]. Therefore, FM should delay the aging of laying hens and redevelop their tissues and organs, especially the brain and reproductive organs.

### 4.2. Serological Indices Regulate the FM Process

The process of FM in laying hens is a complex process of neurohormonal regulation, which involves stress, disease resistance, and neuromodulation in different stages [43,44,45,46]. The hypothalamic–ovarian–gonadal axis secretes a variety of sex hormones that play a vitally important role in the regulation of reproductive traits in chickens [47,48]. It has been reported that female animals are more tolerant to starvation stress than males, and female mice secrete a large amount of estradiol to maintain energy metabolism and physiological balance under starvation [49]. Furthermore, the steroid hormone E2 can induce apoptosis in placental and ovarian cells in animals, including humans [50,51]. In our study, E_2_ levels were significantly reduced (*p* < 0.05) under starvation stress conditions (2-vs-3) and then gradually increased when feed and water supply was restored to the hens (3-vs-5). TSH can promote thyroid secretion and release of T4, whereas GH [52,53] and T4 [54,55,56] play an important role in the control of reproductive tract development in hens and chicken embryo health. In this study, GH and TSH levels in the recovery period (3-vs-4 and 4-vs-5, respectively) of molting were significantly increased to restore the health status and reproductive potential of hens.

### 4.3. Aging-Related Gene Expression Level Associated with the FM Process

The transcriptome data of the hypothalamus and ovary during the five stages of laying hens showed that gene expression in the hypothalamus and ovary changed significantly before and after the FM process, and the patterns were somewhat similar.

The expression of DEGs did not change significantly in 1-vs-2. Although this period lasted quite a long time (232 days), this slow process of change mainly involved physiological phenomena, such as aging of skeletal muscle and feathers and a decline in ELR. However, laying hens showed hunger stress under the external stimulation of loss of water and feed during 2-vs-3. Although the duration of this process was relatively short (13 days), there were several DEGs during this period, indicating that drastic changes had taken place in laying hens. This process caused rapid feather loss, rapid weight loss, and ELR to quickly drop to zero as ovarian atrophy occurred. The reproductive function of laying hens was shut down in a short time. During the recovery period of molting, chickens were gradually supplied feed and water, and their physical strength gradually recovered, which contributed to laying hens recovering weight, feathers, and ELR. However, the recovery period of molting lasted for a relatively longer time. After 31 days of recovery (3-vs-4), the ELR recovered to nearly half (47.2%), and the ELR recovered to the SPEP (87.3%) for another 27 days (4-vs-5). Thus, ELR increased steadily during the period of 3-vs-5, and the reproductive system function of laying hens was gradually restored as the hens redeveloped. 

Another interesting phenomenon was that gene expression levels in the hypothalamus and ovary were similar in periods of 1, 2, and 5, which indicates that FM made laying hens recover to their initial state of physical fitness. We then focused on changes in gene expression level during natural aging (1-vs-2), forced molt aging (2-vs-3), and forced molt recovery (3-vs-5).

### 4.4. Physical Changes Are Slow in the Early Natural Aging Process

The group 1-vs-2 presented ELR that was downgraded from the first peak (0.941, period I) to 0.774 (period II) under natural aging conditions. This process takes a long time and mainly involves the slow aging of chickens, accompanied by a gradual decline in ELR and plumage coverage. However, life activities were relatively stable, and a high ELR was maintained during this process. Therefore, the GO terms of DEGs in the hypothalamus were enriched in thyroid gland development, response to heat, and embryonic skeletal system morphogenesis. Heat stress has been shown to decrease egg production and shell quality, ultimately causing significant economic losses to the poultry industry [57], as it not only affects the quality of chicken meat traits [58] but also leads to disease in severe cases [59]. The GO terms of DEGs in the ovary were mainly enriched in the immune response to maintain the basic egg-laying function. KEGG analysis of DEGs in the ovary revealed enrichment in cytokine–cytokine receptor interaction and toll-like receptor signaling pathway, and both pathways are involved in the pathogenic process of avian pathogenic *Escherichia coli* infection of respiratory epithelial cells [60].

### 4.5. FM Accelerates Aging and Redevelopment of Hens 

We focused on the functional enrichment of DEGs during the molting implementation period (2-vs-3) and molting recovery periods (3-vs-5). Among them, there were more common DEGs in these two periods than in other periods, indicating that some common biological pathways are activated in the two reversible processes of FM.

Some immune pathways were enriched in DEGs of 2-vs-3 in both the hypothalamus (immune response, antigen processing, and presentation of endogenous peptide antigen via MHC class I and antigen processing and presentation) and ovary (wound healing and immune response). Additionally, laying hens also activate the self-repair mechanism to adapt to this special stress environment, and we also found GO terms of negative regulation of appetite in DEGs of the hypothalamus. Furthermore, we found some apoptotic signaling pathways (negative regulation of neuronal apoptotic process, extrinsic apoptotic signaling pathway, and mitotic cell cycle arrest) in DEGs of 2-vs-3 in the ovary, which eventually hindered cell growth and differentiation, causing ovarian development to stagnate and atrophy.

During the 3-vs-5 period, the DEGs in the hypothalamus were enriched in growth factor activity, neuropeptide hormone activity, multicellular organism development, cartilage development, granulosa cell differentiation, and extraocular skeletal muscle development. This is because the hypothalamus is a prominent nerve center that regulates visceral and endocrine activity [61]. The DEGs in the ovary were mainly enriched in calcium ion-regulated exocytosis of neurotransmitters, noncanonical Wnt signaling pathways, multicellular organism development, cellular response to hormone stimulus, response to progesterone, and structural constituent of muscle. Wnt signaling is closely related to early embryonic development and ovary formation in chickens [62,63,64]. Ovarian tissue redeveloped and the secretion of progesterone increased, which stimulated the differentiation of ovarian granulosa cells, thereby restarting the reproductive performance of laying hens and restoring their ELR. Moreover, DEGs of the 3-vs-5 periods in the hypothalamus were also enriched in the TGF-β signaling pathway, which plays critical roles in embryogenesis and adult tissue homeostasis by regulating cell proliferation, differentiation, migration, and apoptosis [65,66]. 

Additionally, we found GO terms in DEGs of the 2-vs-3 period for NADH dehydrogenase (ubiquinone) activity and mitochondrial respiratory chain complex I both in the hypothalamus and ovary. The NADH dehydrogenase subunit 2 gene is a mitochondrial gene involved in age-related signaling pathways and is implicated in premature aging syndromes [67]. The aging accumulation of mtDNA mutations can impair the oocyte NADH/NAD+ redox state in the mitochondrial membrane, thereby reducing the fertility of female mammals [68,69]. This is also an important reason for the difficulty older women have in conceiving. KEGG for oxidative phosphorylation was enriched in DEGs of both the hypothalamus and ovary during the two reversible biological processes, whereas mitochondrial oxidative phosphorylation decreased during cell aging and thereby affected cell metabolism [70]. Moreover, oxidative stress is the main cause of ovarian aging in high-producing hens after 480 days of age [71]. Therefore, FM accelerates cell aging stress and redevelopment to realize the rejuvenating process of laying hens.

### 4.6. Dynamic Expression Patterns of Aging-Related Genes Are Consistent with the ELR 

STEM analysis showed that the pattern of gene expression in profile 1 (881 genes) closely matched the pattern of ELR for the five periods. Among them, 45 genes were involved in cell and organ aging when their expression was inhibited. These genes cause the aging of tissues or organs of chickens under starvation stress, but they restarted the development of tissues and organs after stress exposure and restored the reproductive potential of hens.

GO terms of DEGs in profile 1 showed that FM of chicken is a complex biological process including stress response (G-protein-coupled receptor signaling pathway), development (extraocular skeletal muscle development and negative regulation of BMP signaling pathway), reproduction (granulosa cell differentiation), and aging (NADH dehydrogenase (ubiquinone) activity). G-protein-coupled receptors are key cell-surface proteins that can transform extracellular environmental change signals into intracellular biochemical signals, causing changes in intracellular energy metabolism and secretion of hormones to adapt to the external stress environment [72]. The BMP signaling pathway plays an important role in the development of the aging skeleton [73,74]. Of course, the DEGs of profiles 1 and 13 were all annotated in the KEGG pathway of oxidative phosphorylation related to cell aging [75]. Therefore, it was confirmed again that these DEGs might mainly affect the physical fitness of hens by regulating cell aging, apoptosis, and development during FM, thereby indirectly changing ELR. 

### 4.7. Aging-Related Hub Genes Were Identified by WGCNA 

In our previous research, we separated the hypothalamus and ovary of hens to study their roles in the FM process. However, it was reported that high rates of egg production of hens could be comprehensively regulated through the hypothalamic–pituitary–ovarian axis [76,77,78]. Furthermore, WGCNA has many distinct advantages over other methods because the analysis focuses on the association between coexpression modules and phenotypic traits, and the results have high reliability and biological significance [79]. Therefore, we next put hypothalamus and ovary data together to perform WGCNA. ELRs during five different periods were defined as phenotypic traits. Genes in the same module are functionally related to each other. WGCNA allows for the identification of biologically relevant modules and hub genes that may eventually serve as biomarkers for regulation. 

Ultimately, five significant hub genes (*INO80D, HELZ, AGO4, ROCK2,* and *RFX7*) in the brown module were identified. *INO80D* can accelerate atherosclerotic aging and induce hypertension and cataracts [80]. *HELZ* is associated with various mRNA decay factors, regulating the expression of genes related to the development of the nervous system [81]. *AGO4* is closely related to ovarian development in laying hens, and its mutation may induce ovarian cancer [82]. Inhibition of *ROCK2* expression can promote autophagy in cardiomyocytes under the induction of starvation and senescence, thus slowing aging [83], and *ROCK2* in chickens can regulate ovarian follicular development through the hypothalamic–pituitary–ovarian axis [84]. *RFX7* can maintain the basic function of natural killer cells and improve immune defense mechanisms in animals [85].

## 5. Conclusions

In our study, to reveal dynamic gene expression patterns of the hypothalamus and ovary during FM in five growth periods in chickens, we first performed pairwise comparison of RNA-seq analysis for the five periods using the hypothalamus and ovary. The results showed there were more DEGs in the three periods 1-vs-2, 2-vs-3, and 3-vs-5 than in other periods, while the reversible biological process of cell aging and redevelopment existed in the two periods 2-vs-3 and 3-vs-5. STEM analysis was performed on all DEGs from the three periods for the hypothalamus and ovary, and 45 genes were involved in cell aging and 8 genes were involved in autophagy during FM. Finally, five significant brown module genes were identified by WGCNA, among which *INO80D* and *ROCK2* were related to cell aging, and the other three genes were related to hunger stress and ovarian development. Therefore, the process of FM of laying hens can cause dynamic changes in gene expression levels related to cell aging and redevelopment, thus leading to dynamic changes in ELR.

## Figures and Tables

**Table 1:** Description of chicken population information before and after molting in five different sampling periods.

Period	Days of Age	Egg Production	Numbers of Hens	Egg-Laying Rate	Description
1	224	41,460	44,079	0.941	first peak of egg production
2	456	31,180	40,228	0.774	preparation period for molting
3	469	99	39,992	0.002	cessation of stress period
4	500	18,770	39,752	0.472	recovery period of molting
5	527	34,630	39,677	0.873	second peak of egg production

## Data Availability

All the RNA-seq datasets are deposited in the NCBI Short Read Archive under the accession number PRJNA725045.

## Supplementary Materials

The following are available online at https://www.mdpi.com/article/10.3390/genes12111767/s1, Table S1. Average concentration of serological indices during five periods, Table S2. Summary statistics for sequence quality of 30 samples, Table S3. DEGs between 10 groups in the hypothalamus and ovary, Table S4. DEGs whose expression trends were consistent with ELR were related to aging, autophagy or apoptosis, and development, Table S5. Top five genes in the network of the top 100 brown genes ranked by the MCC method, Figure S1. Significant GO enrichment of DEGs (*p* < 0.05) for comparison of 1-vs-2 in the hypothalamus. BP (Biological Process), CC (Cell Component), MF (Molecular Function), Figure S2. Significant GO enrichment of DEGs (*p* < 0.05) for comparison of 1-vs-2 in the ovary. BP (Biological Process), CC (Cell Component), MF (Molecular Function), Figure S3. Significant GO enrichment of DEGs (*p* < 0.05) for comparison of 2-vs-3 in the hypothalamus, Figure S4. Significant GO enrichment of DEGs (*p* < 0.05) for comparison of 2-vs-3 in the ovary. BP (Biological Process), CC (Cell Component), MF (Molecular Function), Figure S5. Significant GO enrichment of DEGs (*p* < 0.05) for comparison of 3-vs-5 in the hypothalamus. BP (Biological Process), CC (Cell Component), MF (Molecular Function), Figure S6. Significant GO enrichment of DEGs (*p* < 0.05) for comparison of 3-vs-5 in the ovary. BP (Biological Process), CC (Cell Component), MF (Molecular Function), Figure S7. Determination of the soft threshold (β=8). The left panel shows the analysis of scale-free fit index and the right panel refers to the analysis of mean connectivity.
