# Supplementary material for: Transcriptomic Analysis of Laying Hens Revealed the Role of Aging-Related Genes during Forced Molting
Source: Genes (Basel). 2021 Nov 7;12(11):1767. doi: 10.3390/genes12111767 (PMC8621152; doi:10.3390/genes12111767)
Supplement: Supplementary file 1 [file genes-12-01767-s001.zip › Supplementary Information.pdf]

Supplementary information

- **Supplementary Table S1** Average concentration of serological indices during five periods
- **Supplementary Table S2** Summary statistics for sequence quality of 30 samples
- **Supplementary Table S3** DEGs between 10 groups in the hypothalamus and ovary
- **Supplementary Table S4** DEGs whose expression expression trends were consistent with ELR were related to aging, autophagy or apoptosis, development
- **Supplementary Table S5** Top five genes in the network of the top 100 brown genes ranked by the MCC method
- **Supplementary Figure S1** Significant GO enrichment of DEGs ($P < 0.05$) for comparison of 1-vs-2 in the hypothalamus. BP (Biological Process), CC (Cell Component), MF (Molecular Function).
- **Supplementary Figure S2** Significant GO enrichment of DEGs ($P < 0.05$) for comparison of 1-vs-2 in the ovary. BP (Biological Process), CC (Cell Component), MF (Molecular Function).
- **Supplementary Figure S3** Significant GO enrichment of DEGs ($P < 0.05$) for comparison of 2-vs-3 in the hypothalamus.
- **Supplementary Figure S4** Significant GO enrichment of DEGs ($P < 0.05$) for comparison of 2-vs-3 in the ovary. BP (Biological Process), CC (Cell Component), MF (Molecular Function).
- **Supplementary Figure S5** Significant GO enrichment of DEGs ($P < 0.05$) for comparison of 3-vs-5 in the hypothalamus. BP (Biological Process), CC (Cell Component), MF (Molecular Function).
- **Supplementary Figure S6** Significant GO enrichment of DEGs ($P < 0.05$) for comparison of 3-vs-5 in the ovary. BP (Biological Process), CC (Cell Component), MF (Molecular Function).
- **Supplementary Figure S7** Determination of the soft threshold (β=8). The left panel shows the analysis of scale-free fit index and the right panel refers to the analysis of mean connectivity.

Supplementary Table S1 Average concentration of serological indices during five periods

Serological Indices	1	2	3	4	5
E(pg/mL)	53.41 ± 9.55^{ab}	35.54 ± 7.34^{a}	53.79 ± 6.01^{b}	47.32 ± 15.27^{ab}	33.86 ± 18.35^{ab}
FSH(mIU/ml)	6.22±0.78	5.58±2.03	5.65±0.76	5.11±1.16	6.06±1.85
T4(ng/mL)	171.3±45.91	195.72±32.07	191.0±22.52	228.85±13.68	169.58±38.95
CT(pg/mL)	45.37±4.49	50.40±1.51	41.55±7.98	36.99±12.48	41.99±10.24
GH(ng/mL)	15.18 ± 2.76^{ab}	15.48 ± 4.10^{ab}	12.77 ± 2.79^{a}	18.34 ± 1.80^{b}	16.98 ± 3.66^{ab}
PROG(pmol/L)	1004.89±84.05	1151.87±361.95	1102.88±378.04	1155.23±199.43	1107.32±306.62
TSH(mU/L)	9.97 ± 2.11^{ab}	11.12 ± 2.94^{ab}	11.85 ± 2.34^{ab}	9.33 ± 1.13^{a}	13.16 ± 1.46^{b}

Note: Values in the same row with the same or no letter superscripts mean no significant difference ($p > 0.05$), while with different small letter superscripts mean significant different ($p < 0.05$).

Supplementary Table S2 Summary statistics for sequence quality of 30 samples

Samples	Raw Reads (10^6)	Raw bases (10^6)	Clean Reads (10^6)	Clean bases (10^6)	Q20 (%)	Q30 (%)	GC content (%)
1-1-o	48.71	7355.73	45.58	6883.05	97.22	92.84	51.99
1-1-h	44.88	6776.65	42.11	6359.27	97.06	92.39	49.78
1-2-o	45.95	6937.83	42.88	6475.32	97.22	92.75	50.72
1-2-h	44.93	6784.03	42.10	6357.15	96.91	92.09	49.86
1-3-o	48.27	7288.34	45.18	6822.24	97.01	92.36	51.33
1-3-h	43.15	6515.45	40.38	6097.26	97.12	92.58	49.83
2-1-o	46.43	7011.22	43.35	6545.67	97.17	92.59	50.31
2-1-h	49.69	7503.31	46.57	7031.88	97.13	92.6	50.01
2-2-o	42.92	6480.30	40.16	6063.80	96.95	92.15	50.38
2-2-h	47.44	7164.17	44.44	6711.19	97.12	92.56	49.99
2-3-o	43.25	6531.05	40.43	6104.54	97.18	92.61	50.44
2-3-h	48.43	7312.23	45.61	6886.87	97.14	92.62	49.93
3-1-o	40.87	6130.03	37.91	5686.19	97.68	94.24	50.54
3-1-h	41.72	6258.21	38.75	5812.03	97.55	93.96	49.25
3-2-o	42.48	6372.62	39.22	5882.57	97.16	92.55	49.39
3-2-h	44.55	6682.44	41.36	6203.45	97.62	94.01	49.33
3-3-o	38.63	5794.97	35.95	5392.27	97.69	94.21	49.53
3-3-h	41.55	6232.85	38.54	5781.74	97.41	93.59	49.48
4-1-o	43.33	6498.75	40.40	6060.07	96.97	92.73	50.21
4-1-h	50.82	7622.77	47.42	7113.36	96.67	92.11	49.64
4-2-o	46.53	6979.16	43.48	6522.42	96.91	92.51	49.81
4-2-h	53.53	8029.17	49.99	7498.31	96.81	92.39	49.76
4-3-o	46.65	6997.53	43.60	6539.85	97.03	92.67	49.46
4-3-h	51.22	7682.71	48.03	7204.12	96.77	92.25	49.48
5-1-o	53.25	7987.22	49.03	7353.82	97.72	93.96	47.86

5-1-h	47.07	7060.75	43.73	6558.82	97.65	93.84	47.48
5-2-o	49.82	7472.76	46.11	6916.56	97.72	93.94	47.58
5-2-h	44.44	6666.10	41.34	6200.93	97.41	93.38	47.39
5-3-o	50.34	7551.63	46.18	6926.70	97.35	93.3	48.14
5-3-h	45.91	6886.34	42.70	6404.44	97.51	93.58	47.28

Note: i-j-o (i=1,2,3,4,5; j=1,2,3) represent ovary samples from 3 biological replicas in 5 periods;
i-j-h (i=1,2,3,4,5; j=1,2,3) represent hypothalamus samples from 3 biological replicas in 5 periods.

Supplementary Table S3 DEGs between 10 groups in the hypothalamus and ovary

Period	Tissue	Down-regulated genes	Up-regulated genes	Total DEGs
1-vs-2	hypothalamus	71	113	184
	ovary	173	200	373
1-vs-3	hypothalamus	310	1179	1489
	ovary	842	1688	2530
1-vs-4	hypothalamus	197	344	541
	ovary	380	808	1188
1-vs-5	hypothalamus	68	118	186
	ovary	577	431	1008
2-vs-3	hypothalamus	158	932	1090
	ovary	326	875	1201
2-vs-4	hypothalamus	99	226	325
	ovary	35	143	178
2-vs-5	hypothalamus	13	10	23
	ovary	40	26	66
3-vs-4	hypothalamus	486	100	586
	ovary	420	153	573
3-vs-5	hypothalamus	941	124	1065
	ovary	721	161	882
4-vs-5	hypothalamus	217	74	291
	ovary	152	41	193

Supplementary Table S4 DEGs whose expression trends were consistent with ELR were related to aging, autophagy or apoptosis, development

Genes function (Numbers)	Genes and corresponding references
cell aging (45)	*ABCB8*[1], *BMP8A*[2], *BOLA3*[3], *CCK*[4], *CDKN1C*[5, 6], *CFD*[7], *COX5A*[8], *FAM171A2*[9], *FBXO46*[10], *FGFR4*[11, 12], *FMC1*[13], *FOXC1*[14], *FOXL2*[15, 16], *FZD5*[17], *GGCX*[18], *HIF3A*[19], *HNRNPA0*[20], *IGFBP4*[21], *MANF*[22], *MRPS12*[23], *MTERF4*[24], *NAT8B*[25], *NEDD8*[26], *NOG*[27], *NOG2*[28], *PABPN1*[29], *POLD4*[30], *POLE4*[31], *PPP1R1B*[32], *RAD9A*[33], *RBM8A*[34], *RPL35A*[35], *SELENOH*[36], *SEMA6C*[37], *SNCB*[38], *SOCS1*[39, 40], *SREBF1*[41], *SST*[42], *SUV39H1*[43],*TAP1*[44], *TCN2*[45], *TINAGL1*[46], *UCP3*[47, 48], *ZAR1*[49], ZC3H10[50]
cell autophagy or apoptosis (8)	*ANKRD39*[34], *CBLN4*[34], *GRN* [51], *PAXX* [52], *PPIL1*[53], *S100A16*[54], *SCYL1*[55], *TGFB1*[56]
Cell development (12)	*ACP5*[57], *ADAMTS4*[58, 59], *CARTPT* [60, 61], *CDK5R1*[62], *CDK5R2*[63], *FJX1*[64], *GTPBP3*[65], *H2AFV* [66], *HSD17B1*[67], *RENBP* [68], *VSTM2L* [69], *WBP1*[70]

Supplementary Table S5 Top five genes in the network of the top 100 brown genes ranked by the MCC method

Rank	Name	Score	Description
1	*INO80D*	38	INO80 complex subunit D
2	*HELZ*	10	helicase with zinc finger
3	*AGO4*	2	argonaute RISC component 4
3	*ROCK2*	2	Rho associated coiled-coil containing protein kinase 2
3	*RFX7*	2	regulatory factor X7

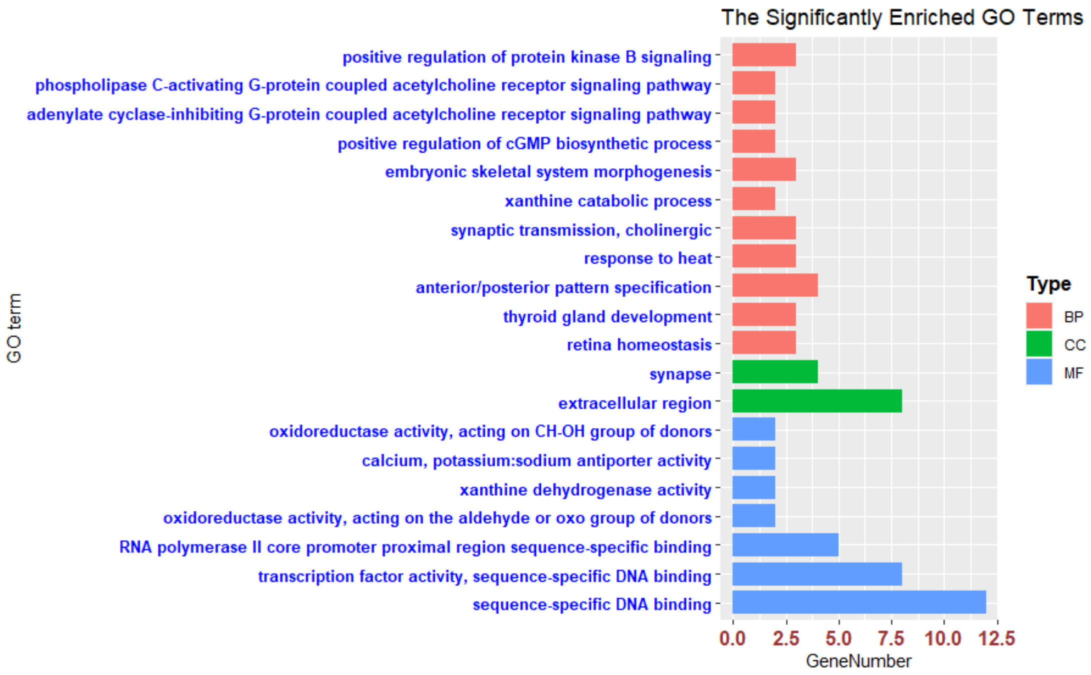

Supplementary Figure S1 Significant GO enrichment of DEGs ($P < 0.05$) for comparison of 1-vs-2 in the hypothalamus.

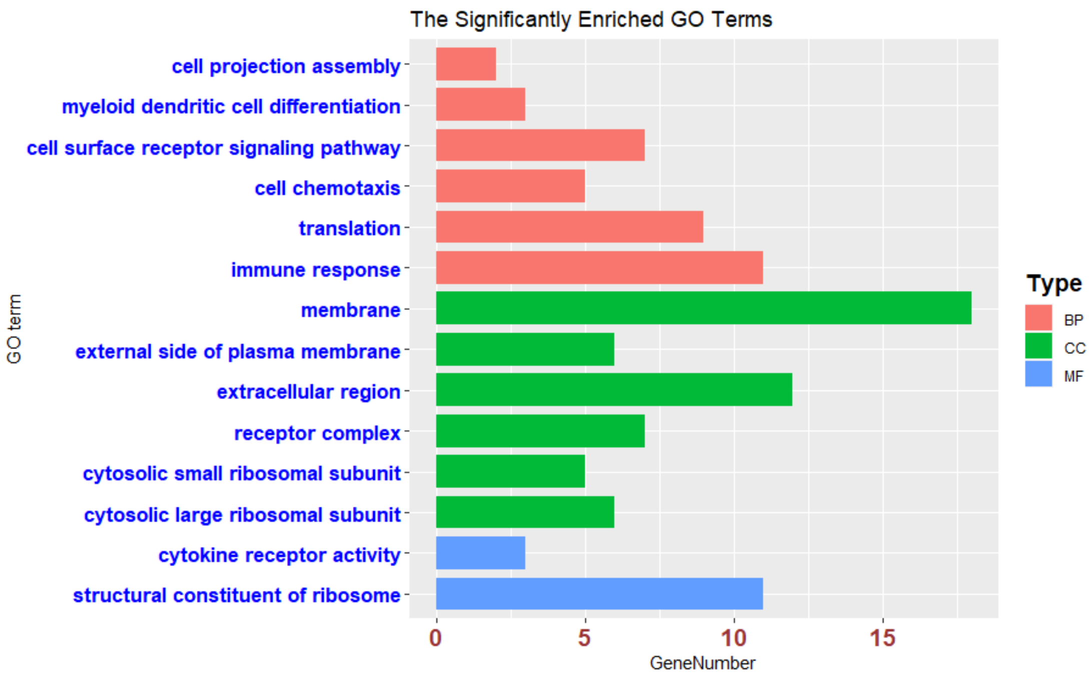

Supplementary Figure S2 Significant GO enrichment of DEGs (P < 0.05) for comparison of 1-vs-2 in the ovary.

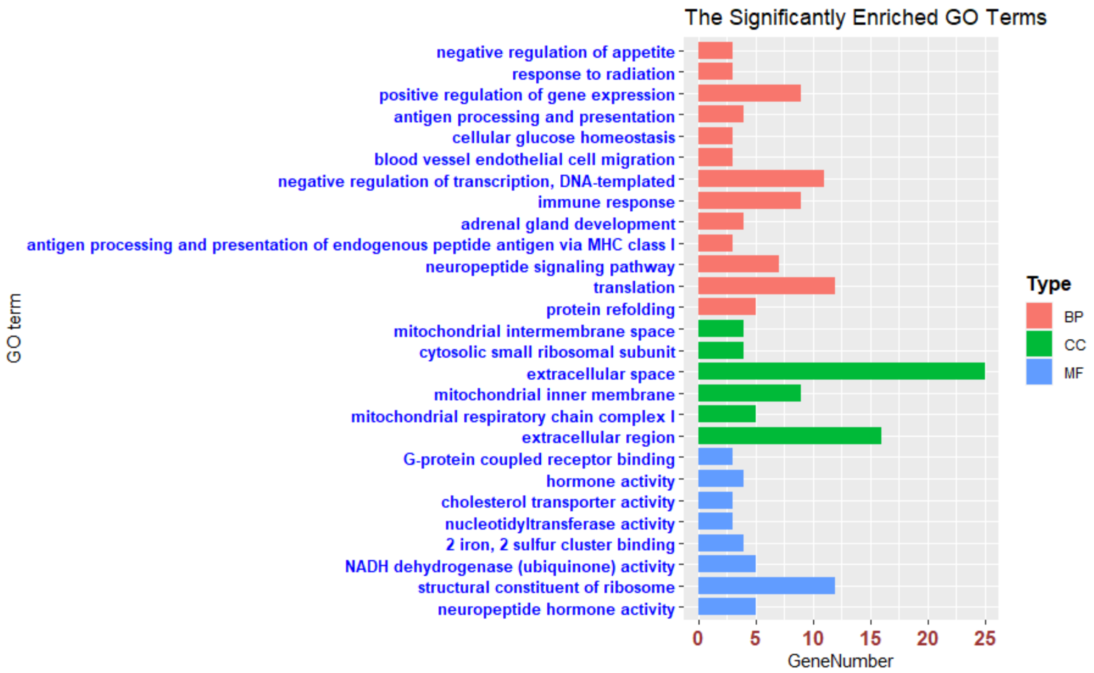

Supplementary Figure S3 Significant GO enrichment of DEGs ($P < 0.05$) for comparison of 2-vs-3 in the hypothalamus.

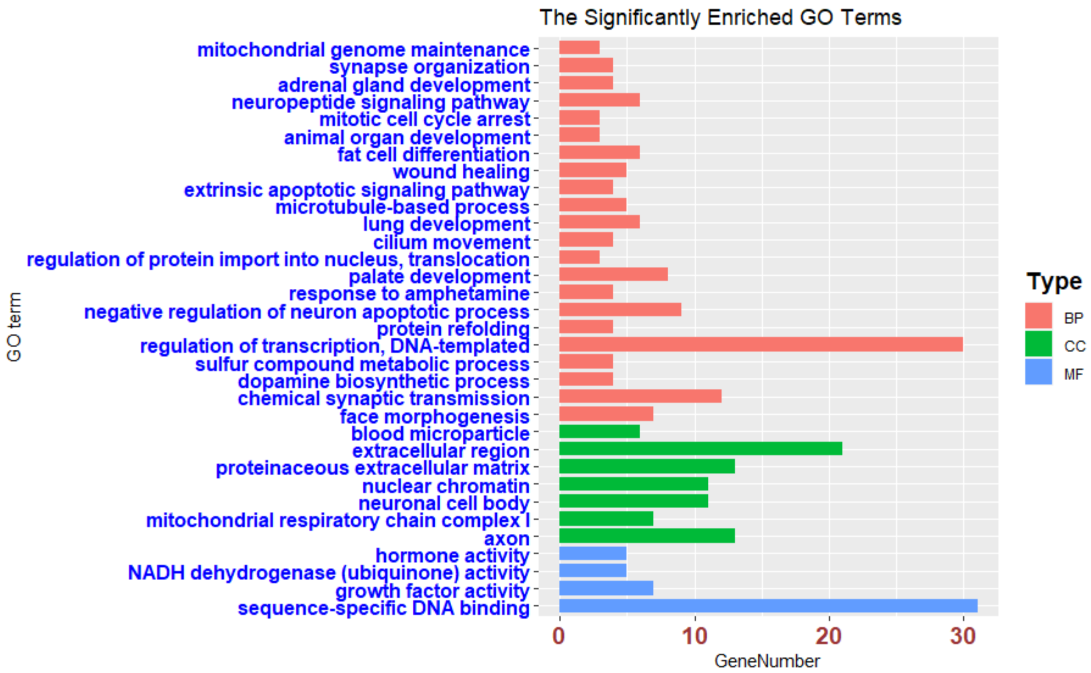

Supplementary Figure S4 Significant GO enrichment of DEGs ($P < 0.05$) for comparison of 2-vs-3 in the ovary.

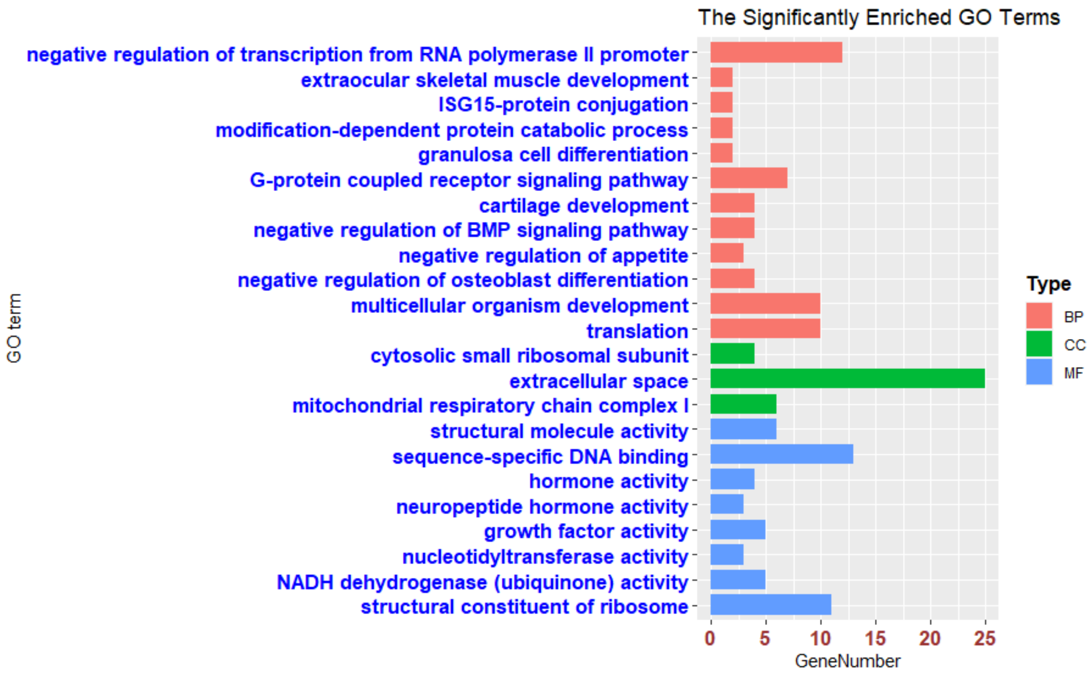

Supplementary Figure S5 Significant GO enrichment of DEGs ($P < 0.05$) for comparison of 3-vs-5 in the hypothalamus.

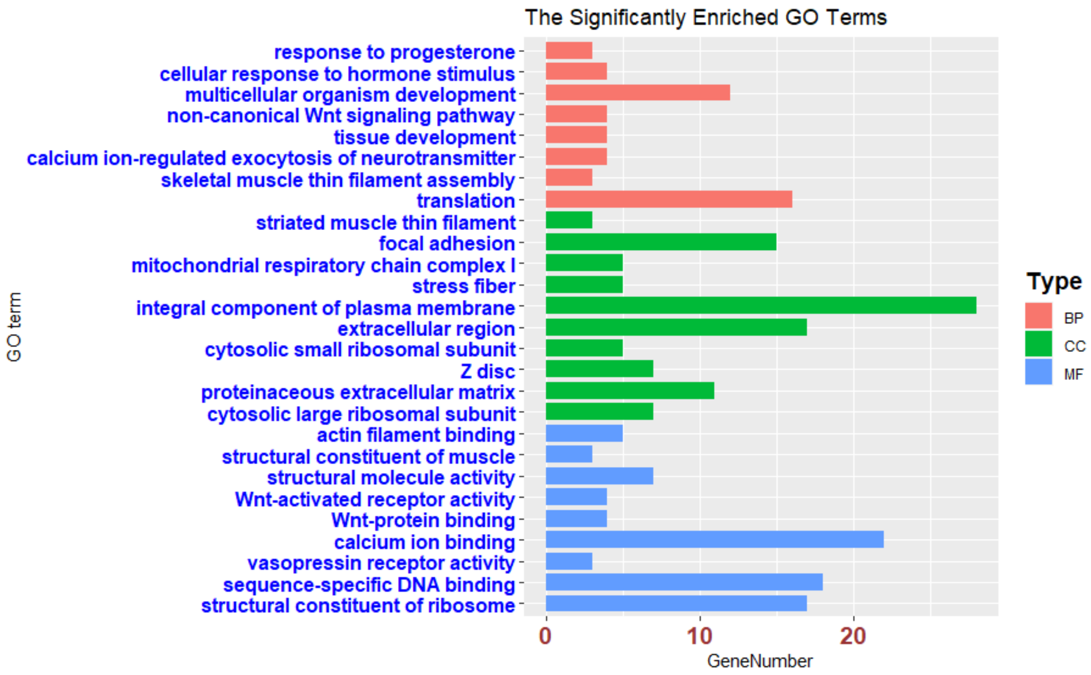

Supplementary Figure S6 Significant GO enrichment of DEGs ($P < 0.05$) for comparison of 3-vs-5 in the ovary.

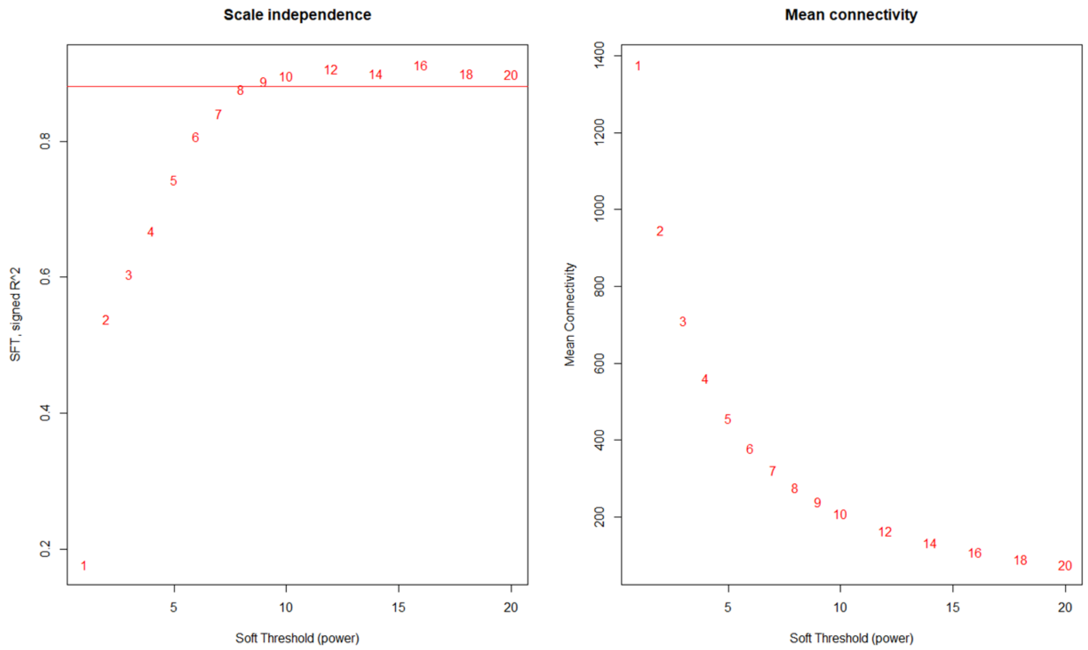

Supplementary Figure S7 Determination of the soft threshold (β=8). The left panel shows the analysis of scale-free fit index and the right panel refers to the analysis of mean connectivity.

References

[1] Liesa M, Qiu W, Shirihai OS, Shirihai. Mitochondrial ABC transporters function: the role of ABCB10 (ABC-me) as a novel player in cellular handling of reactive oxygen species. Biochim Biophys Acta 2012; 1823: 1945-57.

[2] Ciller IM, Palanisamy SK, Ciller UA, McFarlane JR. Postnatal expression of bone morphogenetic proteins and their receptors in the mouse testis. Physiol Res 2016; 65: 673-82.

[3] Tajima K, Ikeda K, Chang HY, Chang CH, Yoneshiro T, Oguri Y, et al. Mitochondrial lipoylation integrates age-associated decline in brown fat thermogenesis. Nat Metab 2019; 1: 886-98.

[4] MacIntosh CG, Morley JE, Wishart J, Morris H, Jansen JB, Horowitz M, et al. Effect of exogenous cholecystokinin (CCK)-8 on food intake and plasma CCK, leptin, and insulin concentrations in older and young adults: evidence for increased CCK activity as a cause of the anorexia of aging. J Clin Endocrinol Metab 2001; 86: 5830-7.

[5] Van de Pette M, Tunster SJ, John RM. Loss of Imprinting of Cdkn1c Protects against Age and Diet-Induced Obesity. Int J Mol Sci 2018; 19: 2734.

[6] Liu W, Liu C, Yin B, Peng XZ. Functions of miR-9 and miR-9* during Aging in SAMP8 Mice and Their Possible Mechanisms. Zhongguo Yi Xue Ke Xue Yuan Xue Bao 2015; 37: 253-8.

[7] Ezure T, Sugahara M, Amano S. Senescent dermal fibroblasts negatively influence fibroblast extracellular matrix-related gene expression partly via secretion of complement factor D. Biofactors 2019; 45: 556-62.

[8] Xiyang YB, Liu R, Wang XY, Li S, Zhao Y, Lu BT, et al. COX5A Plays a Vital Role in Memory Impairment Associated With Brain Aging via the BDNF/ERK1/2 Signaling Pathway. Front Aging Neurosci 2020; 12: 215.

[9] Xu W, Han SD, Zhang C, Li JQ, Wang YJ, Tan CC, et al. The FAM171A2 gene is a key regulator of progranulin expression and modifies the risk of multiple neurodegenerative diseases. Sci Adv 2020; 6: eabb3063.

[10] Choppara S, Ganga S, Manne R, Dutta P, Singh S, Santra MK. The SCF(FBXO46) ubiquitin ligase complex mediates degradation of the tumor suppressor FBXO31 and thereby prevents premature cellular senescence. J Biol Chem 2018; 293: 16291-306.

[11] Sasaki N, Gomi F, Yoshimura H, Yamamoto M, Matsuda Y, Michishita M, et al. FGFR4 Inhibitor BLU9931 Attenuates Pancreatic Cancer Cell Proliferation and Invasion While Inducing Senescence: Evidence for Senolytic Therapy Potential in Pancreatic Cancer. Cancers (Basel) 2020; 12: 2976.

[12] Zhang W, Xu Y, Zhang L, Wang S, Yin B, Zhao S, et al. Synergistic effects of TGFβ2, WNT9a, and FGFR4 signals attenuate satellite cell differentiation during skeletal muscle development. Aging Cell 2018; 17: e12788.

[13] Gupta S, Zola H, Brooks DA, Bradley J, Fikrig SM, Mariano E, et al. Monoclonal antibody-defined B cell subsets in aging humans and Down's syndrome. Gerontology 1984; 30: 388-92.

[14] Lay K, Kume T, Fuchs E. FOXC1 maintains the hair follicle stem cell niche and governs stem cell quiescence to preserve long-term tissue-regenerating potential. Proc Natl Acad Sci U S A 2016; 113: 1506-15.

[15] Ottolenghi C, Uda M, Hamatani T, Crisponi L, Garcia JE, Ko M, et al. Aging of oocyte, ovary, and human reproduction. Ann N Y Acad Sci 2004; 1034: 117-31.

[16] Marongiu M, Deiana M, Marcia L, Sbardellati A, Asunis I, Meloni A, et al. Novel action of FOXL2 as mediator of Col1a2 gene autoregulation. Dev Biol 2016; 416: 200-11.

[17] Harada S, Mabuchi Y, Kohyama J, Shimojo D, Suzuki S, Kawamura Y, et al. FZD5 regulates cellular senescence in human mesenchymal stem/stromal cells. Stem Cells 2021; 39: 318-30.

[18] Azuma, K. and S. Inoue, Multiple Modes of Vitamin K Actions in Aging-Related Musculoskeletal Disorders. Int J Mol Sci 2019; 20: 2844.

[19] Chen D, Liu J, Meng J, Li D, Zhao P, Duan Y, et al. Integrative Analysis of Long Non-Coding RNAs (lncRNAs), miRNAs, and mRNA-Associated ceRNA Network in Lung Tissue of Aging Mice and Changes After Treatment with Codonopsis pilosula. Med Sci Monit 2020; 26: e921580.

[20] Lee BP, Pilling LC, Bandinelli S, Ferrucci L, Melzer D, Harries LW. The transcript expression levels of HNRNPM, HNRNPA0 and AKAP17A splicing factors may be predictively associated with ageing phenotypes in human peripheral blood. Biogerontology 2019; 20: 649-63.

[21] Wang H, Zhu X, Shen J, Zhao EF, He D, Shen H, et al. Quantitative iTRAQ-based proteomic analysis of differentially expressed proteins in aging in human and monkey. BMC Genomics 2019; 20: 725.

[22] Neves J, Chirco KR, Cedron-Craft W, Chew S, Zhu J, Jasper H, et al. MANF delivery improves retinal homeostasis and cell replacement therapies in ageing mice. Exp Gerontol 2020; 134: 110893.

[23] Wang G, Shimada E, Zhang J, Hong JS, Smith GM, Teitell MA, et al. Correcting human mitochondrial mutations with targeted RNA import. Proc Natl Acad Sci U S A, 2012; 109: 4840-5.

[24] Spåhr H, Habermann B, Gustafsson CM, Larsson NG, Hallberg BM. Structure of the human MTERF4-NSUN4 protein complex that regulates mitochondrial ribosome biogenesis. Proc Natl Acad Sci U S A 2012; 109: 15253-8.

[25] Rigby MJ, Ding Y, Farrugia MA, Feig M, Cortese GP, Mitchell H, et al. The endoplasmic reticulum acetyltransferases ATase1/NAT8B and ATase2/NAT8 are differentially regulated to adjust engagement of the secretory pathway. J Neurochem 2020; 154: 404-23.

[26] Da Costa IC, Schmidt CK. Ubiquitin-like proteins in the DNA damage response: the next generation. Essays Biochem 2020; 64: 737-52.

[27] Wang C, Oshima M, Sashida G, Tomioka T, Hasegawa N, Mochizuki-Kashio M, et al. Non-Lethal Ionizing Radiation Promotes Aging-Like Phenotypic Changes of Human Hematopoietic Stem and Progenitor Cells in Humanized Mice. PLoS One 2015; 10: e0132041.

[28] Wang X, Chen XJ. A cytosolic network suppressing mitochondria-mediated proteostatic stress and cell death. Nature 2015; 524: 481-4.

[29] Olie CS, Riaz M, Konietzny R, Charles PD, Pinto-Fernandez A, Kiełbasa SM, et al. Deacetylation Inhibition Reverses PABPN1-Dependent Muscle Wasting. iScience 2019; 12: 318-332.
[30] Nicolas, E., E.A. Golemis, and S. Arora, POLD1: Central mediator of DNA replication and repair, and implication in cancer and other pathologies. Gene 2016; 590: 128-41.
[31] Abdelmohsen K, Srikantan S, Tominaga K, Kang MJ, Yaniv Y, Martindale JL, et al. Growth inhibition by miR-519 via multiple p21-inducing pathways. Mol Cell Biol 2012; 32: 2530-48.
[32] Cheong LZ, Sun T, Li Y, Zhou J, Lu C, Li Y, et al. Dietary krill oil enhances neurocognitive functions and modulates proteomic changes in brain tissues of d-galactose induced aging mice. Food Funct 2017; 8: 2038-45.
[33] Mayer RL, Schwarzmeier JD, Gerner MC, Bileck A, Mader JC, Meier-Menches SM, et al. Proteomics and metabolomics identify molecular mechanisms of aging potentially predisposing for chronic lymphocytic leukemia. Mol Cell Proteomics 2018; 17: 290-303.
[34] Zou D, Li R, Huang X, Chen G, Liu Y, Meng Y, et al. Identification of molecular correlations of RBM8A with autophagy in Alzheimer's disease. Aging (Albany NY) 2019; 11: 11673-85.
[35] Mirzaei M, Pushpitha K, Deng L, Chitranshi N, Gupta V, Rajput R, et al. Upregulation of Proteolytic Pathways and Altered Protein Biosynthesis Underlie Retinal Pathology in a Mouse Model of Alzheimer's Disease. Mol Neurobiol 2019; 56: 6017-34.
[36] Zhang L, Zhu JH, Zhang X, Cheng WH. The Thioredoxin-Like Family of Selenoproteins: Implications in Aging and Age-Related Degeneration. Biol Trace Elem Res 2019; 188: 189-95.
[37] Yan W, Zhou S, Shen W, Cheng J, Yuan S, Ye S, et al. Suppression of SEMA6C promotes preantral follicles atresia with decreased cell junctions in mice ovaries. J Cell Physiol 2019; 234: 4934-43.
[38] Hadrian K, Melkonyan H, Schlatt S, Wistuba J, Wasmuth S, Heiligenhaus A, et al. Age-related distribution and potential role of SNCB in topographically different retinal areas of the common marmoset Callithrix jacchus, including the macula. Exp Eye Res 2019; 185: 107676.
[39] Saint-Germain E, Mignacca L, Vernier M, Bobbala D, Ilangumaran S, Ferbeyre G. SOCS1 regulates senescence and ferroptosis by modulating the expression of p53 target genes. Aging (Albany NY) 2017; 9: 2137-62.
[40] Vinod, C. and A. Jagota. Daily Socs1 rhythms alter with aging differentially in peripheral clocks in male Wistar rats: therapeutic effects of melatonin. Biogerontology 2017; 18: 333-45.
[41] Bou Sleiman M, Jha P, Houtkooper R, Williams RW, Wang X and Auwerx J. The Gene-Regulatory Footprint of Aging Highlights Conserved Central Regulators. Cell Rep 2020; 32: 108203.
[42] Kim K and Choe HK. Role of hypothalamus in aging and its underlying cellular mechanisms. Mech Ageing Dev 2019; 177: 74-9.
[43] Zhang YL, Zhao LW, Zhang J, Le R, Ji SY, Chen C, et al. DCAF13 promotes pluripotency by negatively regulating SUV39H1 stability during early embryonic development. Embo j 2018; 37: e98981.

[44] Dong W, Yin X, Sun L, Wang J, Sun S, Zhu G, et al. Age-associated methylation change of TAP1 promoter in piglet. Gene 2015; 573: 70-4.
[45] Kurnat-Thoma EL, Pangilinan F, Matteini AM, Wong B, Pepper GA, Stabler SP, et al. Association of Transcobalamin II (TCN2) and Transcobalamin II-Receptor (TCblR) Genetic Variations With Cobalamin Deficiency Parameters in Elderly Women. Biol Res Nurs 2015; 17: 444-54.
[46] Akaiwa M, Fukui E and Matsumoto H. Matsumoto, Tubulointerstitial nephritis antigen-like 1 deficiency alleviates age-dependent depressed ovulation associated with ovarian collagen deposition in mice. Reprod Med Biol 2020; 19: 50-7.
[47] Kim S, Myers L, Ravussin E, Cherry KE, Jazwinski SM. Single nucleotide polymorphisms linked to mitochondrial uncoupling protein genes UCP2 and UCP3 affect mitochondrial metabolism and healthy aging in female nonagenarians. Biogerontology 2016; 17: 725-36.
[48] Asami DK, McDonald RB, Hagopian K, Horwitz BA, Warman D, Hsiao A, et al. Effect of aging, caloric restriction, and uncoupling protein 3 (UCP3) on mitochondrial proton leak in mice. Exp Gerontol 2008; 43: 1069-76.
[49] Dankert D, Demond H, Trapphoff T, Heiligentag M, Rademacher K, Eichenlaub-Ritter U, et al. Pre- and postovulatory aging of murine oocytes affect the transcript level and poly(A) tail length of maternal effect genes. PLoS One 2014; 9: e108907.
[50] Audano M, Pedretti S, Cermenati G, Brioschi E, Diaferia GR, Ghisletti S, et al. Zc3h10 is a novel mitochondrial regulator. EMBO Rep 2018; 19: e45531.
[51] Upontain S, Sereerak P, Laha T, Sripa B, Tangkawatana P, Brindley PJ, et al. Granulin Expression in Hamsters during Opisthorchis viverrini Infection-Induced Cholangiocarcinogenesis. Asian Pac J Cancer Prev 2018; 19: 2437-45.
[52] Liu X, Shao Z, Jiang W, Lee BJ, Zha S. PAXX promotes KU accumulation at DNA breaks and is essential for end-joining in XLF-deficient mice. Nat Commun 2017; 8: 13816.
[53] Chai G, Webb A, Li C, Antaki D, Lee S, Breuss MW, et al. Mutations in Spliceosomal Genes PPIL1 and PRP17 Cause Neurodegenerative Pontocerebellar Hypoplasia with Microcephaly. Neuron 2021; 109: 241-56.
[54] Wang C, Zhu X, Li A, Yang S, Qiao R, Zhang J. S100A16 regulated by Snail promotes the chemoresistance of nonmuscle invasive bladder cancer through the AKT/Bcl-2 pathway. Cancer Manag Res 2019; 11: 2449-56.
[55] Wang Y, Chen X, Chen X, Chen Q, Huo K. Transcriptional profiling and dynamical regulation analysis identify potential kernel target genes of SCYL1-BP1 in HEK293T cells. Mol Cells 2014; 37: 691-8.
[56] Nüchel J, Ghatak S, Zuk AV, Illerhaus A, Mörgelin M, Schönborn K, et al. TGFB1 is secreted through an unconventional pathway dependent on the autophagic machinery and cytoskeletal regulators. Autophagy 2018; 14: 465-86.
[57] Ren X, Shan WH, Wei LL, Gong CC, Pei DS. ACP5: Its Structure, Distribution, Regulation and Novel Functions. Anticancer Agents Med Chem 2018; 18: 1082-90.
[58] Zhang XH, Qi YX, Gao X, Li JY, Xu SZ. Expression of ADAMTS4 and ADAMTS5 in longissimus dorsi muscle related to meat tenderness in Nanyang cattle. Genet Mol Res 2013; 12: 4639-47.

[59] Boerboom D, Lafond JF, Zheng X, Lapointe E, Mittaz L, Boyer A, et al. Partially redundant functions of Adamts1 and Adamts4 in the perinatal development of the renal medulla. Dev Dyn 2011; 240:1806-14.
[60] Smith GW, Sen A, Folger JK, Ireland JJ. Putative role of cocaine- and amphetamine-regulated transcript (CARTPT) in dominant follicle selection in cattle. Soc Reprod Fertil Suppl 2010; 67: 105-17.
[61] Juengel JL, French MC, Quirke LD, Kauff A, Smith GW, Johnstone PD. Differential expression of CART in ewes with differing ovulation rates. Reproduction 2017; 153: 471-9.
[62] Spreafico M, Grillo B, Rusconi F, Battaglioli E, Venturin M. Multiple Layers of CDK5R1 Regulation in Alzheimer's Disease Implicate Long Non-Coding RNAs. Int J Mol Sci 2018; 19: 2022.
[63] Rademakers R, Sleegers K, Theuns J, Van den Broeck M, Bel Kacem S, Nilsson LG, et al. Association of cyclin-dependent kinase 5 and neuronal activators p35 and p39 complex in early-onset Alzheimer's disease. Neurobiol Aging 2005; 26: 1145-51.
[64] Rock R, Heinrich AC, Schumacher N, Gessler M. Fjx1: a notch-inducible secreted ligand with specific binding sites in developing mouse embryos and adult brain. Dev Dyn 2005; 234: 602-12.
[65] Chen D, Li F, Yang Q, Tian M, Zhang Z, Zhang Q, et al. The defective expression of gtpbp3 related to tRNA modification alters the mitochondrial function and development of zebrafish. Int J Biochem Cell Biol 2016; 77: 1-9.
[66] Madakashira B, Corbett L, Zhang C, Paoli P, Casement JW, Mann J, et al. Variant Histone H2afv reprograms DNA methylation during early zebrafish development. Epigenetics 2017; 12: 811-24.
[67] Yuan X, Zhou X, Qiao X, Wu Q, Yao Z, Jiang Y, et al. FoxA2 and p53 regulate the transcription of HSD17B1 in ovarian granulosa cells of pigs. Reprod Domest Anim 2021; 56: 74-82.
[68] Romao JM, He ML, McAllister TA and Guan LL. Effect of age on bovine subcutaneous fat proteome: molecular mechanisms of physiological variations during beef cattle growth. J Anim Sci 2014; 92: 3316-27.
[69] Liu H, Zhang Z, Zhen P and Zhou M. High Expression of VSTM2L Induced Resistance to Chemoradiotherapy in Rectal Cancer through Downstream IL-4 Signaling. J Immunol Res 2021; 2021: 6657012.
[70] Ortega MS, Kurian JJ, McKenna R and Hansen PJ. Characteristics of candidate genes associated with embryonic development in the cow: Evidence for a role for WBP1 in development to the blastocyst stage. PLoS One 2017; 12: e0178041.
